# Coaxial MoS_2_@Carbon Hybrid Fibers: A Low-Cost Anode Material for High-Performance Li-Ion Batteries

**DOI:** 10.3390/ma10020174

**Published:** 2017-02-13

**Authors:** Rui Zhou, Jian-Gan Wang, Hongzhen Liu, Huanyan Liu, Dandan Jin, Xingrui Liu, Chao Shen, Keyu Xie, Bingqing Wei

**Affiliations:** 1 State Key Laboratory of Solidification Processing, Center for Nano Energy Materials, School of Materials Science and Engineering, Northwestern Polytechnical University and Shaanxi Joint Lab of Graphene (NPU), Xi’an 710072, China; zhourui12@mail.nwpu.edu.cn (R.Z.); liuhongzhennwpu@outlook.com (H.L.); liuhuanyan@mail.nwpu.edu.cn (H.L.); jindandan0210@163.com (D.J.); liuxingrui@nwpu.edu.cn (X.L.); shenchao@nwpu.edu.cn (C.S.); kyxie@nwpu.edu.cn (K.X.); 2 Department of Mechanical Engineering, University of Delaware, Newark, DE 19716, USA

**Keywords:** MoS_2_, composite, anode, low cost, Li-ion battery

## Abstract

A low-cost bio-mass-derived carbon substrate has been employed to synthesize MoS_2_@carbon composites through a hydrothermal method. Carbon fibers derived from natural cotton provide a three-dimensional and open framework for the uniform growth of MoS_2_ nanosheets, thus hierarchically constructing coaxial architecture. The unique structure could synergistically benefit fast Li-ion and electron transport from the conductive carbon scaffold and porous MoS_2_ nanostructures. As a result, the MoS_2_@carbon composites—when serving as anodes for Li-ion batteries—exhibit a high reversible specific capacity of 820 mAh·g^−1^, high-rate capability (457 mAh·g^−1^ at 2 A·g^−1^), and excellent cycling stability. The use of bio-mass-derived carbon makes the MoS_2_@carbon composites low-cost and promising anode materials for high-performance Li-ion batteries.

## 1. Introduction

The worsening environmental problems and energy crisis have accelerated the development of electric vehicles and portable electronics, which forward an ever-growing demand for lithium-ion batteries (LIBs) with high energy density and excellent rate capability [1,2]. Among the LIB components, the commercial anode material of graphite suffers from low theoretical specific capacity (i.e., 372 mAh·g^−1^), slow reaction kinetics, and possible safety issues resulting from its low discharge voltage (<0.2 V) that may cause the formation of lithium dendrites [3,4,5]. Therefore, it is imperative to explore a high-performance anode material that can serve as an alternative replacement of the graphite-based anode [6,7,8]. 

In recent years, two-dimensional (2D) graphene-like nanomaterials such as transition metal dichalcogenides (TMDs) have shown great potential for their applications in energy conversion and storage fields [9,10,11,12]. As a typical TMD, MoS_2_ possesses a layered structure analogous to graphite, and is composed of S-Mo-S layers separated by Van der Waals interactions [13,14,15]. The unique structure is favorable for reversible Li^+^ insertion/extraction, thus rendering high theoretical specific capacity (670 mAh·g^−1^), which is almost double that of graphite’s [16,17]. However, the practical application of MoS_2_ is still hindered by its poor cycling stability and inferior rate performance due to its intrinsically low electronic conductivity and the possible structural destruction during repeated charge–discharge processes [18].

To overcome these drawbacks, combining nanostructured MoS_2_ with conductive and flexible materials has been demonstrated to be an effective approach to improve the electrochemical performance. Carbon materials with large specific surface area and excellent electronic conductivity—such as graphene, carbon nanotubes, carbon nanofibers, carbon spheres, etc.—are considered to be ideal substrates in this respect [15,19,20,21]. MoS_2_/graphene hybrid nanoflowers were developed via a simple hydrothermal method, and delivered a specific capacity of 1150 mAh·g^−1^ after 60 cycles [22]. Single-layered ultrasmall nanoplates of MoS_2_ embedded in carbon nanofibers was used as the anode of LIBs, and showed a good cycling capability of 661 mAh·g^−1^ after 1000 cycles [23]. Compared with pure MoS_2_ counterparts, the improved lithium storage performance can be ascribed to the synergistic effect of carbon and MoS_2_ nanostructures. It should be noted that the fabrication of these carbon substrates is extremely time-consuming and costly, which would limit their widespread implementation. Moreover, non-uniform MoS_2_ aggregations on the carbon backbone is another obstacle to achieving a maximum utilization of active materials [24,25]. Therefore, it remains a big challenge to explore a cost-effective MoS_2_-based anode material with excellent electrochemical performance.

In this work, we report the use of a bio-mass-derived carbon substrate from low-cost natural cotton for subsequent growth of MoS_2_ nanosheets. The carbon fiber substrate provides not only a three-dimensional (3D) and open framework for homogeneous deposition of nanostructured MoS_2_, but also high electrical conductivity to improve the electrochemical reaction kinetics of MoS_2_. The as-prepared MoS_2_@carbon anode material delivers high specific capacity, excellent rate capability, and long cycling stability. Moreover, the bio-mass-derived-carbon simplifies the fabrication process, making the MoS_2_@carbon composite serve as a cost-effective anode material for high-performance LIBs.

## 2. Results and Discussion

**Figure 1:**
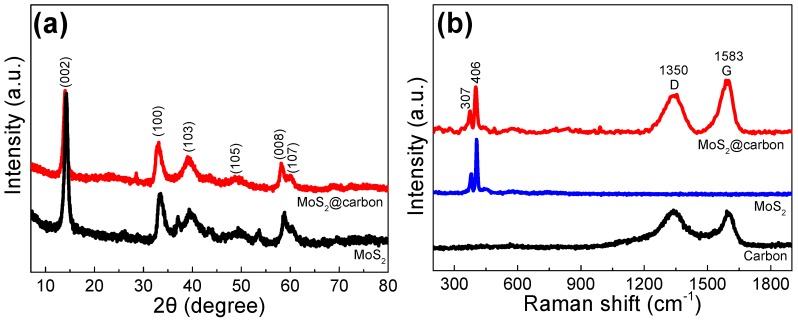
(**a**) XRD patterns of the as-prepared MoS_2_@carbon, pure MoS_2_; (**b**) Raman spectra of the as-prepared MoS_2_@carbon, pure MoS_2_, and the carbon substrate.

The crystalline structure of the as-prepared materials was investigated by X-ray diffraction (XRD). As shown in Figure 1a, both MoS_2_@carbon and pure MoS_2_ exhibit similar XRD profiles. The distinct diffraction peaks with 2θ at around 14°, 33°, 39°, 49°, 59°, and 60° correspond to the (002), (100), (103), (105), (008), and (107) crystal planes of MoS_2_ with 2H phase, respectively [26,27,28]. Raman spectra were recorded to further elucidate the component and structure of the MoS_2_@carbon composite (Figure 1b). It is clearly revealed that the two distinct peaks at around 374 and 406 cm^−1^ can be ascribed to the in-plane E^1^_2g_ and out-of-plane A_1g_ modes of the hexagonal MoS_2_ crystal, respectively [29,30]. In addition, the two broad Raman peaks located at 1350 and 1583 cm^−1^ are readily assigned to the D and G bands of carbon, respectively, indicating the presence of the carbon component in the composite [22,31]. The high I_G_/I_D_ intensity ratio (1.05) reveals good graphitization degree of the carbon substrate, which is favorable for enhancing the electrical conductivity of the MoS_2_/C composites [2,32].

**Figure 2:**
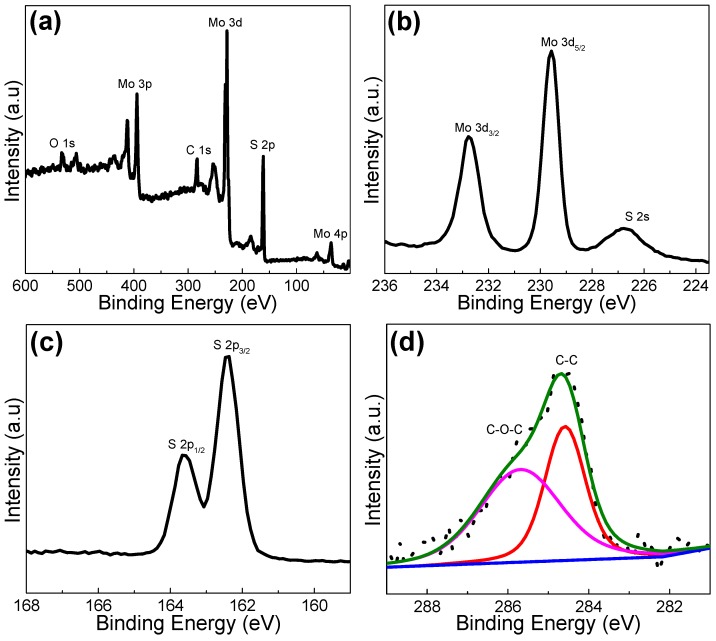
XPS spectra of MoS_2_@carbon composite fiber membranes: (**a**) the survey scan; (**b**) Mo 3d; (**c**) S 2p; (**d**) C 1s.

The composition and chemical states of the as-prepared MoS_2_@carbon were characterized by X-ray photoelectron spectroscopy (XPS). The survey spectrum in Figure 2a indicates the existence of main elements of C, Mo, and S as well as a trace of O element absorbed on the surface [33]. The core level spectrum of Mo 3d (Figure 2b) and S 2p (Figure 2c) show characteristic peaks with binding energies located at 232.6, 229.5, 163.5, and 162.2 eV, which belong to the Mo 3d_3/2_, Mo 3d_5/2_, S 2p_1/2_, and S 2p_3/2_ components of MoS_2_, respectively [34,35]. The C 1s XPS peak (Figure 2d) can be fitted C–C and C–O–C components. All these results reveal the successful preparation of MoS_2_ on the carbon fiber [23].

**Figure 3:**
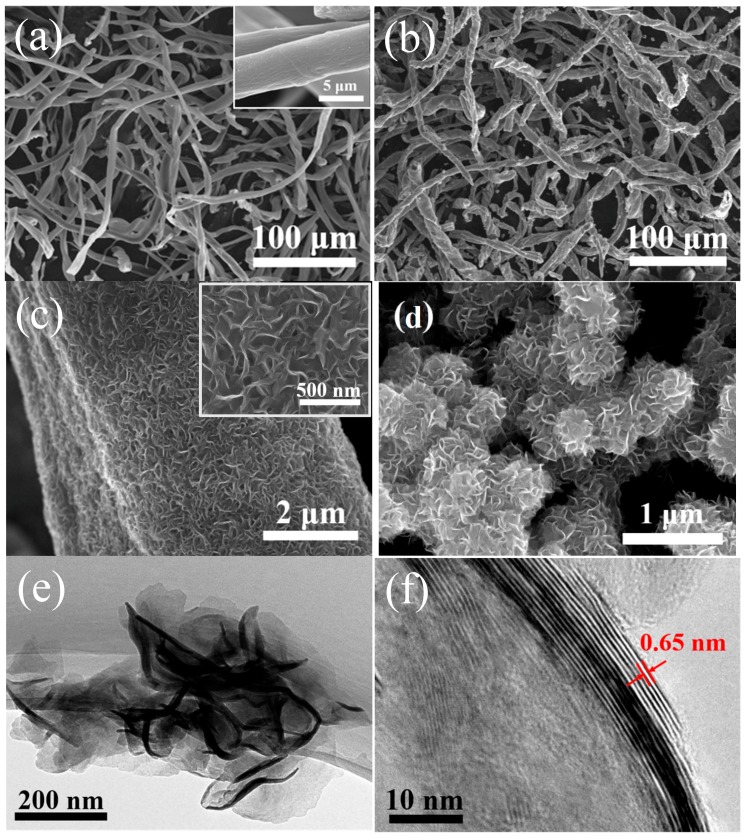
(**a**) Field emission scanning electron microscopy (SEM) image of pure cotton-derived carbon; (**b**,**c**) SEM images; (**e**) high-magnification transmission electron microscopy (TEM) image; and (**f**) high-resolution TEM (HRTEM) image of MoS_2_@carbon; (**d**) SEM image of pure MoS_2_.

Figure 3 shows the field-emission scanning electron microscopy (SEM) and transmission electron microscopy (TEM) images of the pure carbon and MoS_2_@carbon composites. Figure 3a exhibits the typical morphology of pure cotton-derived carbon substrate, which is composed of one-dimensional (1D) fibers with smooth surface (inset) and an average diameter of 7 μm. In addition, the carbon fibers form a randomly-entangled 3D network, which is favorable for solution flux and thus uniform growth of MoS_2_. Figure 3b shows a panoramic view of the MoS_2_@carbon composites, which inherit the 1D fibrous morphology of carbon substrates. Figure 3c shows the uniform and conformal deposition of MoS_2_ nanostructures on the carbon surface, thus constructing coaxial configuration. The higher-resolution SEM image (inset in Figure 3c) reveals porous MoS_2_ nanosheets aligned vertically on the fiber surface. The 1D/2D hierarchical architecture of the MoS_2_@carbon composites is beneficial for electrolyte penetration, and enlarges electrode/electrolyte interface area. The TEM image in Figure 3e exhibits ultrathin nanosheet-like MoS_2_ structures [36]. The high-resolution TEM (HRTEM) image in Figure 3f shows a typical edge view of the MoS_2_ nanosheets with clear lattice fringes. The thickness of the MoS_2_ nanosheets is estimated to be about 10 nm, and the interlayer spacing of 0.65 nm corresponds to the (002) plane of the MoS_2_ [37]. For comparison, pure MoS_2_ nanoflowers with an average diameter of 500 nm were prepared as a control sample (Figure 3d).

**Figure 4:**
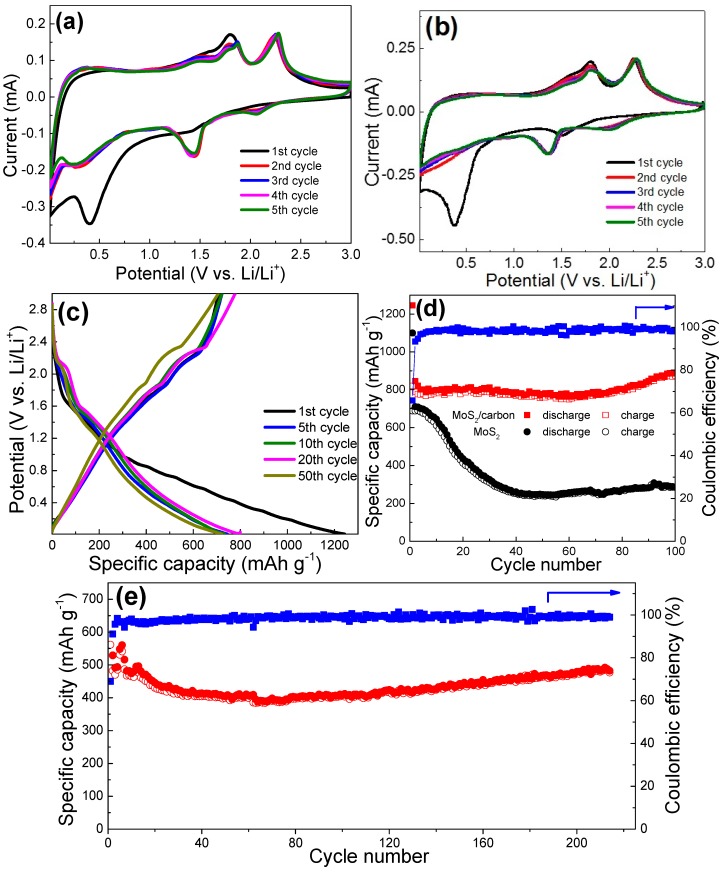
Cyclic voltammetry (CV) curves of the (**a**) MoS_2_@carbon and (**b**) pure MoS_2_ electrodes at 0.1 mV·s^−1^; (**c**) charge–discharge curves of the MoS_2_@carbon at 100 mA·g^−1^. Cycling performance and the coulombic efficiencies of the electrodes tested at (**d**) 100 mA·g^−1^ and (**e**) 2000 mA·g^−^^1^.

To evaluate electrochemical properties, the MoS_2_@carbon composite was investigated as an anode material for lithium-ion batteries. As shown in Figure 4a,b, the MoS_2_@carbon composite electrode shares identical CV curve shapes to pure MoS_2_ electrodes for the initial five cycles at a scanning rate of 0.1 mV·s^−1^ in the potential range of 0.01–3.0 V (versus Li/Li^+^). Specifically, in the first cycle, there are two cathodic peaks appearing at about 1.5 V and 0.35 V. The broad peak at about 1.5 V corresponds to Li^+^ intercalation into the layered structure of the MoS_2_ to form Li*_x_*MoS_2_. The 0.35 V peak is attributed to the further conversion reaction from Li*_x_*MoS_2_ into metallic Mo and Li_2_S [18,31,38]. In the reverse anodic scan, the oxidation peaks at 1.8 V and 2.3 V are associated with the stepwise oxidation of Mo into Mo^4+^/Mo^6+^ and the oxidation of Li_2_S into sulfur or polysulfides, respectively [39]. In the subsequent cycles, the reduction peaks at 1.4 V and 2 V are ascribed to the reduction of Mo^6+^/Mo^4+^ and the formation of Li_2_S, respectively. Galvanostatic charge/discharge cycling measurements were carried out to investigate the lithium storage capacity of the MoS_2_@carbon composites. As shown in Figure 4c, two voltage plateaus at 1.5 V and 0.35 V are observed in the first discharge curve, indicating the two-step lithiation reaction of MoS_2_. In the following discharge curves, the plateaus observed in the first discharge shift towards the higher potentials of 2 V and 1.4 V due to the improved reaction kinetics after the first lithiation. During the charging process, the MoS_2_@carbon composite shows two potential plateaus around 1.8 V and 2.3 V, which are consistent with the CV curves [22,40]. Figure 4d presents the cyclic performance of the as-prepared MoS_2_@carbon composites and pure MoS_2_ powders at a current density of 100 mA·g^−^^1^. It can be seen that the MoS_2_@carbon anode delivers an initial discharge and charge capacities of 1246 and 820 mAh·g^−1^, respectively, which are higher than those of the pure MoS_2_ electrode (i.e., 1100 and 689 mAh·g^−1^) and comparable to the reported MoS_2_/graphene (i.e., 1160 and 896 mAh·g^−^^1^) [41]. The initial capacity loss is mainly caused by the irreversible processes such as the inevitable formation of a solid–electrolyte interface (SEI) film on the electrode surface and some side reactions between Li^+^ and active materials [42,43,44,45,46]. The Coulombic efficiency is as high as 67.6% in the first cycle (vs. 62.6% for the pure MoS_2_), which increases to >98% from the second cycles. More importantly, the composite anode retains a high specific capacity of 869 mAh·g^−1^ after 100 cycles, indicating excellent cycling stability. For comparison, the specific capacity of the pure MoS_2_ electrode decreases significantly to only 290 mAh·g^−1^ after 100 cycles. In addition, the good cyclic performance of the MoS_2_@carbon composite is further confirmed by another cell tested at a current density of 2000 mA·g^−1^. As shown in Figure 4e, the composite electrode can deliver a reversible specific capacity of about 480 mAh·g^−1^ after 210 cycles, which is still higher than the commercial graphite anode (372 mAh·g^−1^).

**Figure 5:**
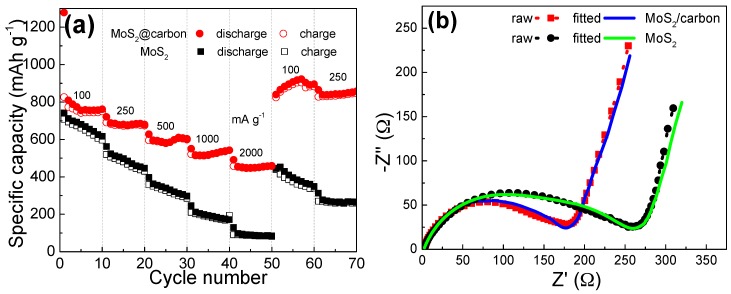
(**a**) Rate performance; and (**b**) electrochemical impedance spectra (EIS) of the MoS_2_@carbon and MoS_2_ powders.

In addition to the higher specific capacity and cyclic performance, the MoS_2_@carbon anode also exhibits enhanced rate capability compared to the pure MoS_2_ powder. Figure 5a compares the rate performance of the anodes. When the current density increases gradually from 100 to 2000 mA·g^−1^, the MoS_2_@carbon anode can maintain a reversible specific capacity of 457 mAh·g^−1^, revealing its excellent rate capability. As the current density returns to 100 and 250 mA·g^−1^, the composite anode recovers its average specific capacities of 891 and 840 mAh·g^−1^, again indicating the outstanding reversibility of the MoS_2_@carbon anode. In sharp contrast, the MoS_2_ powder anode merely delivered about 85 mAh·g^−1^ at 2000 mA·g^−1^. The better rate capability of the MoS_2_@carbon anode can be ascribed to the conductive carbon substrate, which offers high conductivity for rapid electron collection and transfer. A better understanding of the resistive behavior is gained from the electrochemical impedance spectra (EIS). Figure 5b shows the resulting Nyquist plots fitted by Zview software. The EIS curves of both electrodes are composed of one semicircle in the high-to-mediate frequency region and a spike in the low-frequency region. The semicircle corresponds to the charge transfer resistance (*R*_ct_), and the spike is associated with the Li-ion diffusion into the electrode [41]. Notably, the MoS_2_@carbon anode shows a smaller *R*_ct_ than the pure MoS_2_ anode (175 vs. 260 Ω). The decreased *R*_ct_ reveals that the combination of carbon and MoS_2_ components renders improved reaction kinetics for fast electrochemical reactions.

Combining the high specific capacity, good rate capability, and excellent cycling stability, the MoS_2_@carbon composite could serve as a low-cost anode material with attractive electrochemical properties. The performance enhancement of the composite could be attributed to the unique hierarchical coaxial configuration [42,47]. First, the bio-mass-derived 1D carbon fiber substrate offers high conducting pathways for fast electron transfer, while the MoS_2_ nanostructures shorten the electron transport distances. In addition, the 3D scaffold and the porous MoS_2_ nanosheets facilitate smooth electrolyte penetration and ion transport. Both the enhanced electron and ion transport would accelerate the reaction kinetics and thus improve the electrochemical utilization of the composite. Second, the nanoscaled size and uniform distribution of the MoS_2_ nanosheets on the carbon fibers enlarge the contact area between MoS_2_ and electrolyte, thereby offering more active sites for Li-ion storage. Third, the coverage of MoS_2_ on carbon can help to reduce the carbon–electrolyte interaction and reduce side reactions responsible for the irreversible capacity. Fourth, the carbon matrix provides a solid backbone to prevent MoS_2_ nanosheets from restacking and agglomeration during the charge/discharge processes, which ensures the structural integrity of the whole electrode for long-term cycling endurance.

## 3. Materials and Methods 

### 3.1. Synthesis of the MoS_2_@Carbon Composites

All reagents were of analytical grade and used as received without further purification. In a typical procedure, natural cotton was employed as a biomass source of the carbon substrate. The cotton was first carbonized at 900 °C for 3 h (heating rate: 5 °C/min) under N_2_ atmosphere in a horizontal tube furnace to obtain carbon. Subsequently, MoS_2_@carbon composites were synthesized using the one-step hydrothermal method. Typically, 0.076 g of ammonium molybdate tetrahydrate ((NH_4_)_6_Mo_7_O_24_·4H_2_O, AMT) and 1 g of thiourea was dissolved in 35 mL deionized water under magnetic stirring for 30 min. Then, 100 mg of the carbon substrate was immersed into the solution, which was transferred into a 50 mL Teflon-lined stainless steel autoclave and kept at 180 °C for 24 h. After cooling down to room temperature, the black product was washed with deionized water and ethanol several times, and finally dried at 60 °C for 12 h. MoS_2_@carbon was calcined at 800 °C for 2 h under N_2_ atmosphere to increase the degree of crystallinity. For comparison, MoS_2_ powder was synthesized via the same hydrothermal procedure but without the addition of carbon.

### 3.2. Material Characterization

The phase structure of the samples was characterized by X-ray diffraction (XRD) with Cu Kα radiation (λ = 0.15418 nm) (X’Pert Pro MPD, Philips, Almelo, The Netherlands). Raman spectral analysis was carried out on a Renishaw inVia Raman Spectrometer (Renishaw Invia RM200, London, UK) using an excitation wavelength of 532 nm. The as-prepared sample was placed on conductive resin to observe the morphology using field emission scanning electron microscopy (FE-SEM, FEI Nano SEM 450, FEI, Portland, OR, USA). The composite was dispersed in ethanol under ultrasonication for transmission electron microscopy (TEM, FEI Tecnai F30G^2^, FEI, Portland, OR, USA) investigation. The surface elements and chemical states of the sample were examined by X-ray photoelectron spectroscopy (XPS, ESCALAB 250Xi, Thermo Scientific, Waltham, MA, USA).

### 3.3. Electrochemical Measurements

The electrochemical performance was evaluated using CR 2016 coin cells assembled in an argon-filled glove box. The working electrode was fabricated by casting a slurry of 70 wt % active material, 20 wt % conductive agent (carbon black), and 10 wt % binder (polyvinylidene fluoride) in N-methyl-2-pyrrolidinone (NMP) on a copper foil. Li foil, a Celgard 2400 microporous polypropylene membrane, and a solution of 1M LiPF_6_ in ethylene carbonate (EC), dimethyl carbonate (DMC), and ethylmethyl carbonate (EMC) (1:1:1, volume ratio) served as the counter electrode, the separator, and the electrolyte, respectively. Solartron electrochemical workstation (1260 + 1287, Bognor Regis, UK) was employed to obtain the cyclic voltammetry (CV) in the potential range of 0.01–3.0 V (vs. Li/Li^+^) at a rate of 0.1 mV·s^−1^, and the electrochemical impedance spectra (EIS) in the frequency range from 1 MHz to 50 mHz. Galvanostatic charge/discharge cycling was conducted on a Land battery system (LAND, BT2013A, Wuhan, China) under different current densities.

## 4. Conclusions

A low-cost bio-mass-derived carbon material was explored as effective substrates for the uniform growth of MoS_2_ nanosheets via a hydrothermal approach. The MoS_2_@carbon composite exhibits well-defined 1D/2D hierarchical coaxial architecture, which is favorable for improving the reaction kinetics and the electrode structure. When evaluated as an anode of LIBs, the MoS_2_@carbon composite delivers a high specific capacity of 820 mAh·g^−1^ at 100 mA·g^−1^, superior rate capability with 457 mAh·g^−1^ retained at a high-rate current of 2000 mA·g^−1^, and excellent cycling stability without noticeable capacity loss after 100 cycles. The remarkable lithium ion storage performance makes the present MoS_2_@carbon composite a promising low-cost anode material candidate for high-performance LIBs.
